# Development and evaluation of a genome‐wide Coffee 8.5K SNP array and its application for high‐density genetic mapping and for investigating the origin of *Coffea arabica* L.

**DOI:** 10.1111/pbi.13066

**Published:** 2019-02-04

**Authors:** Virginie Merot‐L'anthoene, Rémi Tournebize, Olivier Darracq, Vimel Rattina, Maud Lepelley, Laurence Bellanger, Christine Tranchant‐Dubreuil, Manon Coulée, Marie Pégard, Sylviane Metairon, Coralie Fournier, Piet Stoffelen, Steven B. Janssens, Catherine Kiwuka, Pascal Musoli, Ucu Sumirat, Hyacinthe Legnaté, Jean‐Léon Kambale, João Ferreira da Costa Neto, Clara Revel, Alexandre de Kochko, Patrick Descombes, Dominique Crouzillat, Valérie Poncet

**Affiliations:** ^1^ Nestlé R&D Center Tours France; ^2^ IRD, UMR DIADE Montpellier France; ^3^ Nestlé Research, NIHS Lausanne Switzerland; ^4^ Plantentuin Meise Meise Belgium; ^5^ NARO Kituuza Uganda; ^6^ Wageningen University Wageningen The Netherland; ^7^ ICCRI Jember Indonesia; ^8^ CNRA Divo Ivory Coast; ^9^ Université Kisangani Kisangani Democratic Republic of Congo; ^10^ INCA Luanda Angola; ^11^ ClassM Marseille France

**Keywords:** SNP array, single‐nucleotide polymorphism, *Coffea arabica* origin, *C. canephora*, *C. eugenioides*, genetic map

## Abstract

Coffee species such as *Coffea canephora* P. (Robusta) and *C. arabica* L. (Arabica) are important cash crops in tropical regions around the world. *C. arabica* is an allotetraploid (2*n* = 4*x* = 44) originating from a hybridization event of the two diploid species *C. canephora* and *C. eugenioides* (2*n* = 2*x* = 22). Interestingly, these progenitor species harbour a greater level of genetic variability and are an important source of genes to broaden the narrow Arabica genetic base. Here, we describe the development, evaluation and use of a single‐nucleotide polymorphism (SNP) array for coffee trees. A total of 8580 unique and informative SNPs were selected from *C. canephora* and *C. arabica* sequencing data, with 40% of the SNP located in annotated genes. In particular, this array contains 227 markers associated to 149 genes and traits of agronomic importance. Among these, 7065 SNPs (~82.3%) were scorable and evenly distributed over the genome with a mean distance of 54.4 Kb between markers. With this array, we improved the Robusta high‐density genetic map by adding 1307 SNP markers, whereas 945 SNPs were found segregating in the Arabica mapping progeny. A panel of *C. canephora* accessions was successfully discriminated and over 70% of the SNP markers were transferable across the three species. Furthermore, the canephora‐derived subgenome of *C. arabica* was shown to be more closely related to *C. canephora* accessions from northern Uganda than to other current populations. These validated SNP markers and high‐density genetic maps will be useful to molecular genetics and for innovative approaches in coffee breeding.

## Introduction

The *Coffea* genus (Rubiaceae family) is a large genus that currently comprises 125 accepted species (http://www.theplantlist.org/1.1/browse/A/Rubiaceae/Coffea/). Despite the large number of species in the genus, only two are economically important and cultivated on a large scale: *Coffea canephora* Pierre ex Froehner (commonly known as Robusta) and *Coffea arabica* L. (Arabica). Both species occur in the inter‐tropical region of Africa with *C. arabica* being mainly restricted to Ethiopia and *C. canephora* native to West and Equatorial Africa (Davis *et al*., 2006).

*Coffea arabica* is an allotetraploid (amphidiploid; C^a^C^a^E^a^E^a^, 2*n* = 4*x* = 44 chromosomes) resulting from a natural hybridization event estimated to have taken place 0.665 million years ago at the most, between the ancestors of present‐day *C. canephora* (C^a^ subgenome donor) and *Coffea eugenioides* S. Moore (E^a^ subgenome donor) (each with 2*n* = 2*x* = 22) (Yu *et al*., 2011). The relatively recent origin and the self‐fertilization of *C. arabica* certainly contributed to its relatively low genetic diversity compared to diploid *Coffea* species (Lashermes *et al*., 2000). *Coffea arabica* was probably introduced to Arabia (now Yemen) from its Ethiopian origin during the 14th century (Chevalier, 1929) and has been cultivated there for at least five centuries. The human dispersal of Arabica coffee from Yemen to the rest of the world started in the early 18th century and occurred mainly via two genetic lineages: Typica and Bourbon. These lineages gave rise to most of the current commercial Arabica cultivars grown worldwide (Anthony *et al*., 2002) and as such this explains the low genetic diversity among the cultivated forms. The lack of genetic diversity in Arabica breeding lines has been recognized as a significant limitation of the varietal tolerance towards biotic and abiotic stresses (Anthony *et al*., 2002).

The closely related wild species (Crop Wild Relatives: CWR) of *C. arabica* harbour a much greater level of genetic variability. They belong to the secondary gene pool of *C. arabica* such as its progenitor species *C. canephora* (Gomez *et al*., 2009). They also represent an important source of gene variants to broaden the cultivated *C. arabica* genetic base. These wild genetic resources might help in developing novel Arabica cultivars with higher resilience to the ongoing changes in climatic conditions.

The development of new genomic tools can help us explore, more deeply and more precisely, the genomic diversity at intra‐ and inter‐specific levels. Two examples of high‐throughput platforms include next‐generation sequencing (NGS) (Davey *et al*., 2011) and the development of DNA microarrays (Gupta *et al*., 2008). Compared to a whole‐genome sequencing methodology, an SNP array approach provides time‐effective, low‐cost and more straightforward genotyping technology for germplasm screening (You *et al*., 2018; Yu *et al*., 2014).

With its relatively small, diploid, sequenced and annotated genome, *Coffea canephora* is highly compliant with genomic‐based breeding approaches using Genome Wide Association Studies (GWAS). Thanks to the rapid development of genomic resources and the publication of the reference genome (Denoeud *et al*., 2014), third‐generation markers based on single‐nucleotide polymorphisms (SNPs) have gradually been identified and assayed in *Coffea*, particularly in *C. arabica* (Sant'Ana *et al*., 2018; Tran *et al*., 2018).

However, high‐throughput genotyping assays are still needed in order to rapidly characterize the coffee genetic diversity and to evaluate the introgression of different CWRs in a cost‐effective way. These assays would ultimately ensure more efficient and time‐effective breeding programmes. To conduct such programmes, measures must be taken to construct high‐density genetic maps. Such maps have already been initiated for *C. canephora* (Denoeud *et al*., 2014; Lefebvre‐Pautigny *et al*., 2010; Leroy *et al*., 2011), and *C. arabica* (Moncada *et al*., 2016; Pearl *et al*., 2004). Some of these maps were also used to identify QTLs for agronomic and quality‐related traits in *C. canephora* (Leroy *et al*., 2011; Mérot‐L'Anthoëne *et al*., 2014) and *C. arabica* (Moncada *et al*., 2016). However, the use of SNP markers to generate denser maps has been poorly exploited so far.

Here, we are reporting on the development of the Coffee8.5K SNPs array that contains 8580 unique and informative SNPs, covering the whole *Coffea canephora* genome. The genome‐wide distribution and accurate identification (coding vs. non‐coding regions) of the SNPs allowed to generate a high‐quality array. This high‐density SNP array has greatly improved the high‐density genetic maps of *C. canephora*. This array also provides a valuable resource for genetic diversity analyses and for the investigation of genetic relatedness between *Coffea arabica* CWRs.

## Results

The whole‐genome coffee SNP array, Coffee8.5K, was primarily designed to provide an efficient screening of the wild genetic germplasm resources in *Coffea*, with two considerations: SNPs had to (i) provide an adequate representation of the genome diversity based on genotyped mapping populations and diverse germplasm collections and (ii) additionally harbour sufficient allelic variations at the level of the functional genes that control important breeding traits. We define the ‘Discovery Panel’ as the set of samples used to design the Coffee8.5K array; the ‘Diversity Panel’ is the set of samples used to validate the array and perform subsequent genetic analyses; the ‘Mapping Populations’ are the Robusta and Arabica mapping progenies used to construct linkage maps.

### Filtering and SNP statistics

#### SNP selection and array design

Analysing resequencing data generated from both *C. arabica* (five genotypes) and *C. canephora* (twelve genotypes) mapped against the *C. canephora* reference genome (Denoeud *et al*., 2014) led to the selection of 9827 high‐quality SNPs.

The selection of SNPs was based on three main criteria: (i) SNPs from the *C. arabica* Discovery Panel were chosen to be associated with the intra‐subgenome polymorphism and had to be polymorphic between the two parent species of the Arabica mapping population, (ii) SNPs selected from the *C. canephora* Discovery Panel had to be found in at least two genotypes in order to avoid rare and individual‐specific alleles; (iii) both genic and intergenic SNPs, in equivalent numbers, were selected with a minimum distance of 40 kb between SNPs, to provide an accurate representation of the genome without redundancy. The final SNP set also included informative markers associated with 149 genes related to traits of agronomic or organoleptic importance (Table S1).

In the end, our Coffee8.5K array comprised 8580 SNPs that met Illumina‐quality criteria during the manufacturing process and were finally successfully synthesized (Table S3), representing an 87.3% effective conversion rate. For initial data extraction and filtering, SNPs with low GenTrain (<0.6) and GenCall (<0.2) scores were considered as missing data prior to genotype calling. Then, a series of quality control steps was performed to ensure the accuracy of the genotype‐calling process. For a given genotype, the CallRate (i.e. the percentage of scorable SNPs) ranged from 81.7% to 97.7% in the different Mapping and Diversity Panels screened, with an average call of 92.3% in the total 262 investigated samples (Table 1). Genotyping was consistent over the two replicates of the four parents (*C. canephora* BP409, Q121, and *C. arabica* Ar8 and Ar36B) and also between the two SNPs duplicated on the SNP array: the signal and genotyping qualities were efficient and reproducible. Moreover, 42 of the 50 SNPs previously targeted by KASPar assays were successfully synthetized on the Coffee8.5K and used for validation purposes. These 42 SNP‐generated genotypes were 100% concordant with those obtained from the KASPar assay, thereby validating the presence of these SNPs in the coffee genome as well as the SNP detection process itself.

**Table 1:** (a) Utilization and efficiency of the Coffee8.5K array. Evaluation of the Coffee8.5K array for application in the three *Coffea* species (*C. canephora*,* C. arabica* and *C. eugenioides*), genetic diversity assessment in the *C. canephora* panel and (b) genetic mapping in the segregating populations of *C. canephora* and *C. arabica*

(a)									
SNP source	Genomic region	Synthesized loci	*C. canephora*	(*N* = 27)*	*C. arabica*	(*N* = 16)*	*C. eugenioides*	(*N* = 6)
			Scorable (%†)	Polymorphic (%‡)	Scorable (%)	Polymorphic in *Dihaploid Et39* (C^a^/E^a^)	Polymorphic (%)	Scorable (%)	Polymorphic (%)
*C. arabica*		**3050**	**1978 (64.9%)**	**465 (23.5%)**	**1790 (58.7%)**	**383 (21.4%)**	**705 (39.4%)**	**1441 (47.2%)**	**110 (7.6%)**
	Coding	208	168	48	154	66	46	138	16
	Non‐coding	2842	1810	417	1636	317	659	1303	94
*C. canephora*		**5530**	**5087 (92.0%)**	**4977 (97.8%)**	**5034 (91.0%)**	**1270 (25.2%)**	**19 (0.4%)**	**4742 (85.8%)**	**145 (3.1%)**
	Coding	3220	3046	3000	3028	801	14	2912	95
	Non‐coding	2310	2041	1977	2006	469	5	1830	50

Total		**8580**	**7065 (82.3%)**	**5442 (77%)**	**6824 (79.5%)**	**1653 (24.2%)**	**724 (10.6%)**	**6183 (72.1%)**	**255 (4.1%)**
	Coding	3428	3214	3048	3182	867	60	3050	111
	Non‐coding	5152	3851	2394	3642	786	664	3133	144

(b)				
SNP source	Genomic region	Synthesized loci	Segregating in	
			*C. canephora* Mapping progeny* (*N* = 93)	*C. arabica* Mapping progeny* (*N* = 138)
*C. arabica*		**3050**	**158 (5.2%)**	**900 (29.5%)**
	Coding	208	14	69
	Non‐coding	2842	144	831
*C. canephora*		**5530**	**1149 (20.8%)**	**45 (0.8%)**
	Coding	3220	651	25
	Non‐coding	2310	498	20

Total		**8580**	**1307 (15.2%)**	**945 (11%)**
	Coding	3428	665	94
	Non‐coding	5152	642	851

(a) *See Table S2; ^†^The percentage of scorable/used loci to successfully synthesized loci; ^‡^The percentage of polymorphic loci to scorable loci in the species.

(b) *See Table S2.

Bold values represent cumulative values of coding + non‐coding statistics.

#### SNP genomic position and distance between SNPs

**Figure 1:**
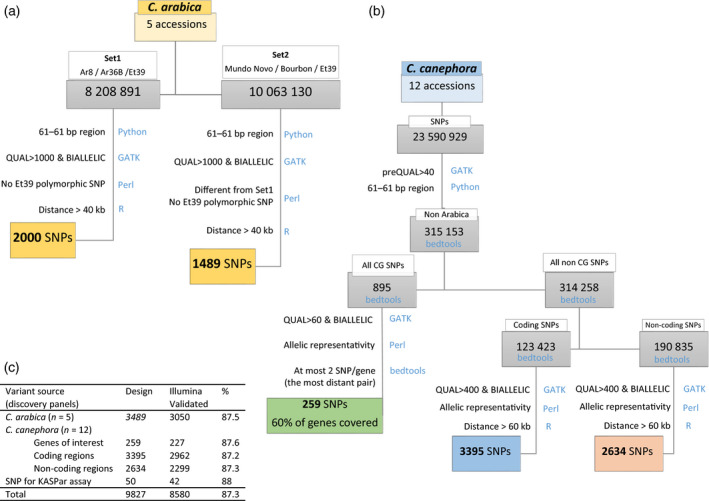
Design and development workflow of the Coffee8.5K array. SNPs markers were identified, filtered and validated from (a) the *C. arabica* or (b) the *C. canephora* Discovery panels. (c) Summary of variant source origin of the Coffee8.5k SNPs. Filters criteria are mentioned together with related tools and programming languages (in blue).

Linkage disequilibrium (LD) occurs when alleles at two or more loci appear together in the same individual more often than would be expected by chance. Two SNPs that are in strong LD provide redundant genotyping information. The requisite knowledge of the LD in the *C. canephora* genome was evaluated over the whole‐genome resequencing dataset of the *C. canephora* Discovery Panel (12 genomes). The linkage disequilibrium declined rapidly across all accessions to half its maximum value at a distance of 8.1 kb (Figure S1). Since this *C. canephora* discovery panel included genotypes from different genetic groups, thereby maximizing the sequence diversity, the LD decayed rapidly. It was particularly higher than when the LD decay was calculated within a single‐genetic group: 17.5 ± 2.7 kb on average over the major genetic groups (min–max: 14.7–21.9 kb) (data not shown). When filtering SNPs with a distance of over 40 kb (Figure 1), we captured the largest proportion of genetic variation. This is due to the fact that the lower the LD between the SNPs is, the more independent information they will represent.

**Figure 2:**
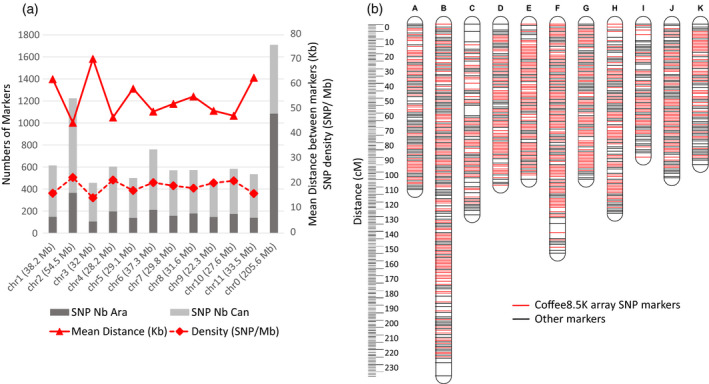
Genetic and genomic distribution of the Coffee8.5K array SNPs. (a) Genome distribution of the 8580 single‐nucleotide polymorphisms (SNPs) synthesized for the array along the 11 pseudo‐chromosomes and the virtual pseudo‐chromosome 0 of unanchored sequences. For each pseudo‐chromosome, we determined: the number of SNPs markers according to their source (*C. arabica*, Ara or *C. canephora*, Can), the mean distance between markers and their density related to the estimated size of the pseudo‐chromosome. (b) *C. canephora* high‐density genetic map of BP409xQ121 progeny, with 11 linkage groups. SNP markers (1307) obtained from the Coffee8.5K array are indicated in red.

In addition, we assessed the quality of coverage by studying the physical distribution of selected Coffee8.5K SNPs along the pseudo‐chromosomes of the *C. canephora* reference genome (Denoeud *et al*., 2014). As shown in Figure 2, the SNPs were widely and evenly distributed along the 11 pseudo‐chromosomes of the genome, no matter which Discovery Panel was used to design them, namely *C. canephora* or *C. arabica*. The average density was 18.8 SNPs per megabase with a mean distance of 54.4 kb between the SNPs (excluding the pseudo‐chromosome 0 made of unanchored sequences).

Lastly, the published annotation of the *C. canephora* reference genome (Denoeud *et al*., 2014; Dereeper *et al*., 2015) was also used to classify the SNPs of the Coffee8.5K array with respect to their position in the genome. A total of 5152 (60%) and 3428 (40%) SNPs were located in non‐coding and coding regions respectively (Table 1). A total of 227 SNPs were located in 149 selected genes of interest (Table S1).

### Coffee8.5K evaluation and usefulness

#### Construction of genetic maps

##### High‐density Robusta genetic map

The Robusta mapping population was derived from an F_1_ cross between BP409 (Congolese hybrid) and Q121 (Conilon‐type derived accession) comprising 93 individuals. After the removal of low‐quality SNPs from the 8580 scorable SNPs, 1307 (15.2%) SNPs were polymorphic between the two parents (BP409 and Q121) and their segregation scored in the Robusta mapping population (Table 2). The majority of these SNPs (80%, 1149) were derived from the *C. canephora* Discovery Panel (Table S2) and 50.9% (665 SNPs) were located in coding regions including 46 SNPs in candidate genes (Table S1). All these polymorphic SNPs were successfully added to the existing markers of the framework linkage map (Denoeud *et al*., 2014), leading to almost double (+43%) the total number of markers on the Robusta map (Table 2). These SNPs were distributed on the 11 linkage groups coded from A to K (Figure 2) and separated by a mean distance of 0.99 cM. However, 76.2% of these markers were clustered (i.e. mapped in groups of markers displaying no recombination) and so finally the polymorphic SNPs were mapped on 620 unique positions separated by a mean distance of 2.1 cM. The clustering of the SNPs could be explained by the small size of the mapping population (93 individuals). Significant segregation distortions (*P* value < 0.01) were observed on 11.4% of the SNPs mapped. The majority (69%) of the strong segregation distortions (*P* value < 0.001) were grouped in two regions located on the LG C (22 SNPs) and LG H (27 SNPs). The final high‐density Robusta genetic map consisted of 3039 loci distributed over 11 linkage groups covering 1370 cM, with a mean distance between markers of 2.2 cM (Table 2). Ninety‐eight percent of the gaps between two adjacent loci were smaller than 2 cM with the largest being 8.9 cM at the distal end of LG B (Figure 2).

**Table 2:** Number of SNP markers added to the Robusta genetic map and coverage in cM of each linkage group in the Robusta linkage maps. The Robusta genetic map based on a F_1_ cross between BP409 (Congolese hybrid) and Q121 (Conilon‐type‐derived accession) comprising 93 individuals. The previous high‐density genetic map has been published by Denoeud *et al*. (2014) with various types of markers (e.g. SSR, RADseq, RFLP)

LG	Total Number of markers	Number of SNPs	%^a^	Coverage (cM)	Mean distance between markers (cM)
A	287	104	36	112	2.6
B	528	220	42	238	2.2
C	194	76	39	129	1.5
D	266	125	47	109	2.4
E	257	125	49	105	2.4
F	341	149	44	155	2.2
G	284	119	42	105	2.7
H	223	108	48	128	1.7
I	158	61	39	90	1.8
J	263	111	42	104	2.5
*K*	238	109	46	95	2.5
TOTAL	3039	1307	43	1370	2.2

a The ratio of SNPs from Coffee8.5K array to total number of loci mapped.

##### Segregation in Arabica mapping progeny

The Arabica F_2_ segregation population (138 individuals) was derived from a cross between two wild Ethiopian Arabicas: Ar8 and Ar36B. We successfully identified 945 polymorphic SNPs between the two parents (Ar8 and Ar36B) (Table 1). As expected, the very large majority (95.2%) of these SNPs were derived from the *C. arabica* Discovery Panel, since they had been selected to discriminate the two wild Ethiopian parents. Based on the *C. canephora* reference genome, these SNPs were evenly distributed along the 11 pseudo‐chromosomes and 41% of them were located on the pseudo‐chromosome 0 (of unanchored sequences). Only 1.3% of the 945 SNPs selected for genetic mapping presented significant segregation distortion (*P* value < 0.01) in the progeny. Consequently, this pool of 945 new SNPs markers provides a promising tool for constructing our Arabica genetic map.

Among the 1307 and 945 SNPs segregating in the Robusta and Arabica mapping populations, respectively, only 46 SNPs (2%) were common and would thus be used for co‐linearity analyses. The mapped SNP markers of the Coffee8.5K SNP array are available in the newly implemented MoccaDB v 2.0 database (Figure S2). This database allows access and visualization of the SNP information, including flanking sequences and possible alleles as well as locations on both the *C. canephora* genome and on the Robusta linkage map.

#### Array polymorphism and its application across *Coffea* species

The 8580 SNP markers on the Coffee8.5K were evaluated for their transferability and genotyping capacity in three *Coffea* species—*C. canephora*,* C. arabica* and *C. eugenioides* Diversity Panels—(Table 1a). Across these three species, 7065 (82.3%) markers successfully genotyped *C. canephora*, 6824 (79.5%) *C. arabica* and 6183 (72.1%) *C. eugenioides*. As expected, we observed a slow decline in the call rate as a function of divergence from the species of the Discovery Panel together with a decrease in polymorphic SNPs. However, the cross‐species application of our array, and particularly the transferability of *C. canephora*‐discovered SNPs to *C. eugenioides,* was shown to be efficient, with a success rate of 85.8% (4742 of the SNPs derived from the *C. canephora* Discovery Panel, Table 1).

The percentage of polymorphic SNPs was much higher for *C. canephora* compared to the *C. arabica* (77% vs. 10.6%). A lower diversity within the allotetraploid, self‐compatible, and recent *C. arabica* species was expected. Because of its alloploidy, homeologous SNPs, that is polymorphism between the two subgenomes C^a^/E^a^, were estimated based on the heterozygous SNPs scored in the dihaploid Et39 (Figure 4b) and represented 24.2% of the scored markers. After filtering for these homeologous markers, we estimated the allelic SNPs (polymorphic positions occurring within a single‐subgenome among individuals) on the 16 tretraploid individuals of the Diversity Panel, which accounted for 10.6% of polymorphic SNPs.

The *C. arabica* neighbour‐joining tree (Figure S3) confirmed the high genetic relatedness of both wild and cultivated individuals, even though the SNP markers were informative enough to discriminate the individuals and this, with high bootstrap levels.

While both *C. canephora* and *C. eugenioides* are outcrossing species, their polymorphism discrepancy (77% vs. 4.1%) is probably due to differences in global genetic diversities of these two species, which present a very different geographical extension (very large for *C. canephora* vs. reduced for *C. eugenioides*). Even if differences in the sampling size (*n* = 24 vs. *n* = 6) may be evoked to explain such a difference, Diversity Panels were chosen on purpose to represent a maximum of the known species diversity.

As a large proportion of the SNPs on the array are Robusta‐derived, filtered to maximize the within *C. canephora* diversity, this may induce an ascertainment bias when other species, such as *C. eugenioides*, are concerned.

#### Characterization of *C. canephora* genetic groups

The capacity of Coffee8.5K to generate genotyping data and to estimate the genetic relatedness among the genotypes was evaluated on the *C. canephora* Diversity Panel (Table S2). Some accessions from this panel had been previously genotyped using 19 microsatellite markers either by (Gomez *et al*., 2009); (Pégard *et al*., 2014) or Nestlé R&D Tours (unpublished). Thus, from these studies, *a priori* classification into the diversity groups was determined (Tables S2 and S4). Additional individuals from Angola and the Democratic Republic of the Congo, with unknown genetic identity, have also been included in this study (Table S2).

**Figure 3:**
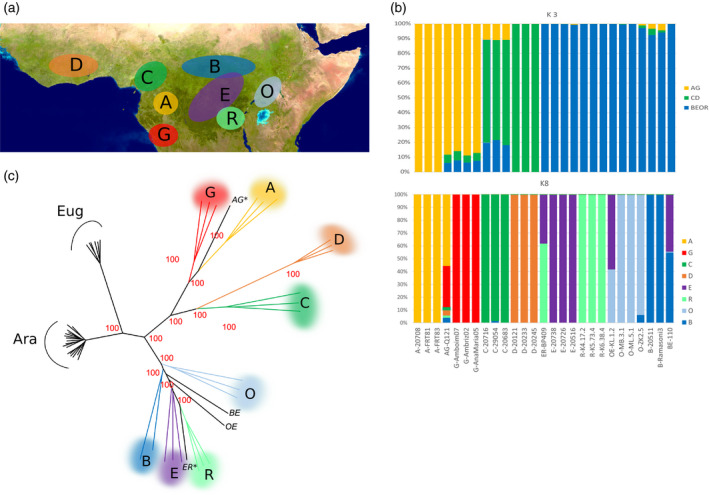
The population structure of the *Coffea canephora* diversity panel (27 accessions). Note that the same colour code is used in all graphs (a) Global distribution of the genetic groups across the *C. canephora* distribution range. Map data from US Dept of State Geographer ©2018 Google Image Landsat/Copernicus Data SIO, NOAA, U.S. Navy, NGA, GEBCO. (b) Population structure analysis using sNMF with three or eight numbers of clusters (*K*). Each colour represents a single cluster, each vertical column represents one accession. (c) A neighbour‐joining tree based on Euclidean distances between the *C. canephora* accessions, together with *C. arabica* and *C. eugenioides* accessions. Individuals from the same group are represented by the same colour, whereas admixed individuals (AG* and EB *, the two parents of the Robusta progeny, and BE, OE) do not have colour.

Genotyping of this Diversity Panel with the Coffee8.5K SNP array resulted in the generation of 4095 polymorphic SNPs without missing data. The population structure and relatedness among accessions was examined using population clustering with sNMF. The genetic structure analysis identified the most likely number of genetic clusters as *K* = 3 (Cluster‐AG, Cluster‐CD and Cluster‐BEOR) (Figure 3b). At higher levels of *K*, new groups emerged revealing a finer subdivision, and at *K* = 8 the accessions were partitioned into eight well‐differentiated groups (A, G, B, C, D, E, O and R), corresponding to different geographic origins (Figure 3a). Among these eight groups, six were confirming the *a priori* classification into the groups A, B, C, D, E and O, whereas two new groups, never previously described, were identified: G (Angola) and R (southern DRC). Three individuals showed mixed ancestry: AG‐Q121, BE‐110, OE‐KL.1.2, and ER‐BP409.

These results were further supported by the neighbour‐joining tree, which distinguished the six known and the two new groups with high bootstrap supports of 100% (Figure 3c). The admixed individuals presented intermediate positions between their ancestral genetic groups. In particular, AG‐Q121 (a hybrid between Groups A and G) and ER‐BP409 (a hybrid between Groups R and E), the two parents of the Robusta mapping progeny, are separated by a maximum genetic distance (at the opposite sides of the tree).

#### Arabica's closest current relatives

**Figure 4:**
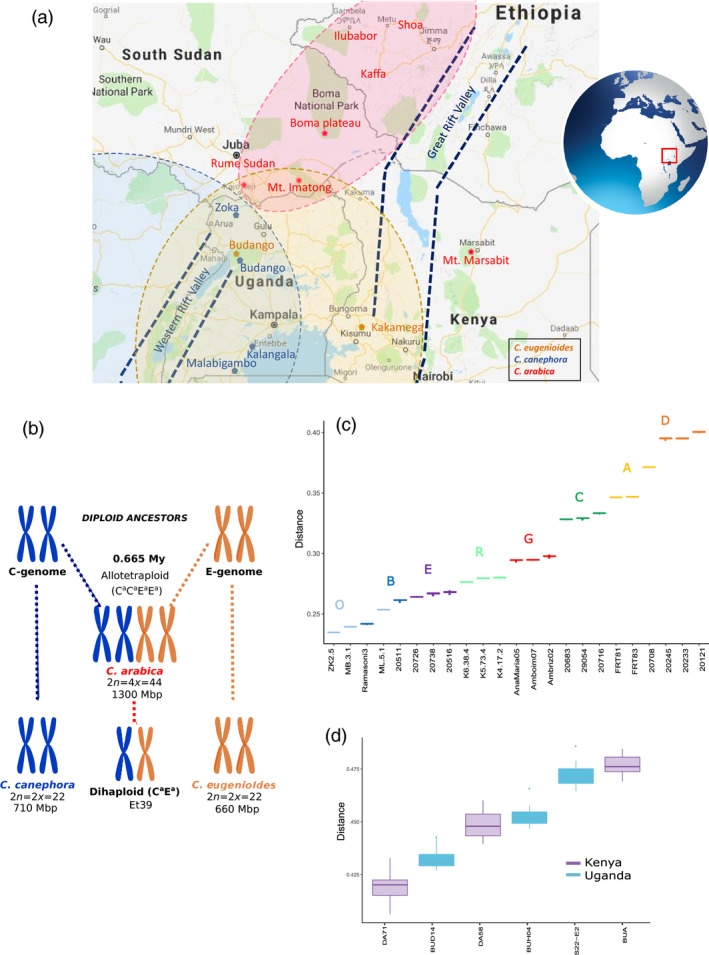
*Coffea arabica* and its progenitor species. (a) Range distribution of the three related species. Dotted lines represent their schematic distribution limit, whereas names with colour labels correspond to sampled sites (*C. canephora* in blue, *C. eugenioides* in gold, and *C. arabica* in red), Map data ©2018 Google, ORION‐ME; (b) Origin of the genomes of the allotetraploid species *Coffea arabica* and of the Dihaploid Et39; (c, d) Haploid Identity‐by‐state distances (IBS) distances between *C. arabica* and accessions of the *C. canephora* with colour code as in Figure 3; and (c) *C. eugenioides* species. The average IBS distances and their standard deviations were calculated over the 17 *C. arabica* individuals.

To identify the putative ancestral *Coffea s*ource populations of each *C. arabica* subgenome, we assigned the *C. arabica* haplotypes to current sampled individuals of the two progenitor species *C. canephora* and *C. eugenioides*. However, because of standard deviation overlaps, the assignation of the Arabica E^a^ subgenome to sampled *C. eugenioides* individuals could not discriminate a specific individual or an origin (Kenya or Uganda) (Figure 4d). On the contrary, since *C. canephora* populations and individuals were well discriminated by our Coffee8.5K array, the *C. arabica* genotypes were differentially assigned to the different genetic groups. In fact, the C^a^ subgenome was more closely related to individuals belonging to Group O (Uganda) or B (Northern DRC) and in particular to an individual of the Zoka forest (Diversity Group O, Northern Uganda) (Figure 4c). Group O encompasses individuals from the Eastern edge of the *C. canephora* distribution; the population from the Zoka Forest (Northern Uganda) is geographically the closest to the current distribution of *C. arabica* (Figure 4a).

## Discussion

Coffee8.5K was designed and developed to assist in mapping *C. canephora* and *C. arabica* and also in assessing the diversity of *C. arabica*,* C. canephora* and other *C. arabica*‐related species (CWRs). The genotypes of the Discovery Panels were chosen to represent a range of common *C. arabica* and *C. canephora* types.

### Fair representation of the genome

The genome size of *C. canephora* is reported to be about 710 Mbp (Noirot *et al*., 2003), with pseudo‐chromosome size ranging from 22.3 Mb (chr9) to 54.5 Mb (chr2) (Denoeud *et al*., 2014). The Coffee8.5K array developed in this study provides complete genome coverage and a fair pseudo‐chromosome representation, with an average density of 18.8 SNPs/Mb and a mean distance between SNPs of 54.4 kb. This density is comparable to other developed SNP arrays in hexaploid wheat (Rimbert *et al*., 2018) (for which the SNP density was one marker every 52 kb on the A‐genome, one every 53 kb on the B‐genome and one every 92 kb on the D‐genome), oil palm (Kwong *et al*., 2016) (one SNP per 11 kb), groundnut (Pandey *et al*., 2017) (1 SNP per 36 kb in A subgenome and 1 SNP per 48 kb in B subgenome) and rice (Yu *et al*., 2014) (12 SNPs per 1 Mb).

Because SNPs located within genic regions have a greater potential to affect gene function and are usually more effective in targeting genes, 40% (3428) of the effective SNPs were selected to be located in genic regions in the Coffee8.5K array.

### Towards high‐density linkage maps

The mapping of 1307 (15.2%) of the Coffee8.5K SNPs on the *C. canephora* map (BP409 X Q121) demonstrates the usefulness of the Coffee8.5K for developing high‐density genetic maps for this species. Combined with previously developed markers (Denoeud *et al*., 2014; Mérot‐L'Anthoëne *et al*., 2014), we obtained a reliable and accurate final map of 3039 markers, including 665 SNPs in coding regions, with only a low fraction of distorted markers. The total map length was 1370 cM with an average distance of 2.2 cM between markers. The length of each linkage group ranged from 238 cM for LG B to 90 cM for LG I. The map obtained in this study is to date the densest map of *C. canephora* and provides access to hundreds of gene‐based markers.

On the contrary, the generation of a high‐density Arabica map is still severely hampered by its allotetraploid nature and the narrow genetic diversity among *C. arabica* accessions. Despite this situation, some mapping efforts have been previously undertaken with AFLP markers (Pearl *et al*., 2004) or more recently with both SSR and SNP markers (Moncada *et al*., 2016). For instance, Moncada *et al*. (2016), after screening 5785 SSR markers, could only find 338 (5.8%) polymorphic markers in the F2 offspring from a cross between *C. arabica* var. Caturra and a wild *C. arabica* accession from Ethiopia. The main difficulty in designing a SNP array useful for *C. arabica* mapping has been detecting co‐dominant SNP markers which were polymorphic within each Arabica subgenome. The parental accessions we selected for our mapping population were two distant wild *C. arabica* accessions, Ar8 and Ar36B, collected in Ethiopia (Guillaumet and Hallé, 1978). By comparing these genotypes, one Bourbon cultivar and a Mundo Novo cultivar (a Bourbon X Typica hybrid) to the Et39 (a spontaneous *C. arabica* dihaploid (Berthaud, 1976; Guillaumet and Hallé, 1978)), our filtering strategy aimed to select within subgenomes polymorphic SNP markers. This approach was especially efficient since it yielded more than 29% (900) of Arabica‐derived SNPs segregating in the mapping progeny and finished with a total of 945 genome‐wide co‐dominant SNP markers.

The high‐density Robusta genetic map generated from this study will not only be useful in ordering future genetic maps such as the one for Arabica, but also in providing a higher marker density than the previous one (Mérot‐L'Anthoëne *et al*., 2014). It will aid in selecting appropriate markers for various molecular breeding applications and in tracking introgression within each linkage group. Markers with even spacing along the genome would be beneficial for generalized use or for identifying marker associations with previously unstudied traits.

Moreover, because our SNP markers are both well‐distributed on the genome and genetically mapped, an additional application of the Coffee8.5K array would be to assist or to validate the genome sequence assembly of both *C. canephora* and *C. arabica* (de Kochko, 2018) by further comparing the genetic position with the physical position.

### Better access to crop wild relative diversity

The Coffee8.5K SNP array was evaluated for its efficiency in studying genetic diversity and genetic relatedness in the Diversity Panel. This set includes representative individuals of the three related species *C. canephora*,* C. eugenioides* and *C. arabica*.

Genotyping data was generated successfully for all three species of the panel with 7065 (82.3%), 6824 (79.5%) and 6183 (72.1%) high‐quality SNP markers in *C. canephora*,* C. arabica* and *C. eugenioides* respectively. This high level of transferability across these species is similar to that previously observed for microsatellite markers (Poncet *et al*., 2004, 2006, 2007). *C. eugenioides* and *C. canephora* belong to different *Coffea* clades (Davis *et al*., 2011; Hamon *et al*., 2017): the East–Central Africa (EC‐Afr) clade and the West and Central Africa (W/C‐Afr) clade respectively (Davis *et al*., 2011). The good transferability of SNP markers discovered from *C. canephora* to the more distantly related species *C. eugenioides* (85.8%), suggests that a high percentage of loci represented on our Coffee8.5K array would also be transferable to other species of the *Coffea* genus, at least from EC‐Afr and W/C‐Afr clades, and represent a substantial improvement in the identification potential of the genetic diversity available within the genetic resources of the *Coffea* wild species.

A high proportion of the tested SNPs (77%) were polymorphic in the *C. canephora* Diversity set. They were also shown to be very efficient for diversity analyses by their application: they allowed to assign each *C. canephora* individual to its *a priori* genetic group—as previously defined by the SSR markers—as well as to discriminate between closely related accessions and to estimate admixture levels. Thanks to these markers, two additional, well‐differentiated genetic groups have been identified and characterized, one from Angola (group G) and the other from DRC (group R). This yielded a whole updated genetic structure of *C. canephora* within eight well‐differentiated and geographically localized groups (see Figure 3 and Table S4).

For *C. arabica*, from the 6824 scored SNPs, 24.2% (1653) turned out to differentiate C^a^ and E^a^ subgenomes in the dihaploid Et39, corresponding to fixed heterozygosity, whereas an estimated 10.6% (724) SNPs represented the within subgenomes allelic polymorphism varying across the *C. arabica* species. Fixed heterozygosity has been suggested to act as a buffer against the reduced population‐level diversity that resulted from the genetic bottleneck associated with polyploid speciation as well as to facilitate the transition to inbreeding (Soltis and Soltis, 2009). The genetic bottleneck undergone by *C. arabica* has been probably severe as suggested by its low within species differentiation, and might have arisen from a single‐polyploidization event (see below).

For the *C. eugenioides* Diversity set, although sharing more than 90% of the called SNP markers with the *C. canephora* set, the polymorphism rate was 19 times lower than for *C. canephora*, certainly due to the lower global genetic diversity of that species. A reduced number of tested *C. eugenioides* individuals can also be evoked, but our sampling covers the available accessions of the species in worldwide collections (Berthaud *et al*., 1980). Furthermore, additional analyses would be challenging since field sampling in the species distribution range has been proven to be difficult.

The Coffee8.5K array has been shown to be especially efficient in fingerprinting individuals within *C. canephora*,* C. eugenioides* and *C. arabica* species and its use by researchers or breeders on larger datasets should provide an opportunity to gain deeper insights into the genetic relatedness among the genotypes and also into the genetic architecture of these important crop wild relative germplasm resources.

### The closest current relatives of Arabica

*Coffea arabica* allotetraploidy resulted from recent natural hybridization between the ancestors of present‐day *C. canephora* (C^a^ genome donor) and *C. eugenioides* (E^a^ genome donor) (Lashermes *et al*., 1999) and is probably the result of a single event (Lashermes *et al*., 2014). *Coffea arabica* displays disomic inheritance with bivalent pairing of homologous chromosomes (Krug and Mendes, 1940), which is in accordance with our present observations in mapping experiments of usual disomic patterns of marker inheritance.

Yu *et al*. (2011) provided evidence of recent *C. arabica* speciation no more than 0.665 million years ago, but also low divergence between the two constitutive subgenomes of *C. arabica* (C^a^ and E^a^) and those of its progenitor species, demonstrating that the nuclear genomes have remained essentially unaltered since the formation of the hybrid.

Using the 17 *C. arabica* accessions, we took advantage of the fact that they exhibited close genetic relationships in order to apply a haploid‐genotype‐based assignation procedure to discover its closest current relatives in our sampling. Using our Coffee8.5K genotyping, we could not preferentially associate the E^a^ subgenome to any of the sampled individuals (Figure 4d).

On the contrary, we were able to preferentially assign the C^a^ subgenome to *C. canephora* of the O (Ugandan) Diversity group (Figure 3) and the shortest distance was observed with a North Ugandan *C. canephora* individual from the Zoka Forest. Geographically, this forest contains the closest *C. canephora* population to the current *C. arabica* distribution (Figure 4a–c).

At the present time, *C. arabica* is mainly found in the southwestern highlands of Ethiopia (Figure 4a), with some occurrence on the Boma plateau in southeastern South Sudan (Thomas, 1942), and on Mount Imantong in Sudan and Mount Marsabit in northern Kenya (Berthaud and Charrier, 1988). *C. arabica* is the main *Coffea* species that occurs in those regions and is geographically isolated from all diploid coffee species in the genus, which includes its two progenitor species *C. canephora* and *C. eugenioides*. Moreover, *C. arabica* also differs from *C. canephora* in terms of current environmental requirement and predicted niche distribution (Gomez *et al*., 2016). Thus, precise localization in Africa of the cradle of *C. arabica*, based on the present distribution of its two progenitor species appears difficult.

Two non‐exclusive scenarios could be suggested for the origin and geographical isolation of *C. arabica*. (i) Upon hybridization, *C. arabica* could have followed one of the typical patterns of the polyploid distribution with peripheral expansion outside the range of the distribution of its diploid parental species. Indeed, it has been suggested that plants with double genomes—auto‐ or allo‐polyploids—have the potential to develop phenotypic novelties, increase their adaptability and obtain higher fitness features that would render them more tolerant towards changing conditions than their diploid counterparts (Amborella Genome *et al*., 2013). This was particularly well illustrated with the survival and proliferation of polyploid plant lineages during the Cretaceous–Tertiary mass extinction event (Fawcett *et al*., 2009). Following the hypothesis that hybridization may favour establishment in novel habitats (Pillon *et al*., 2009; Rieseberg *et al*., 2007), *C. arabica* could have migrated northwards—away from the overlapping distribution range of *C. canephora* and *C. eugenioides—*towards its current distribution range with specific environmental requirement (Gomez *et al*., 2016). Its original population is genetically related to the current Zoka population, which thus represents the southernmost remnant of the original distribution. (ii) Meanwhile, considering that the constitution and the extent of tropical forests have varied considerably during the late Quaternary period (Mumbi *et al*., 2008), past *C. canephora* and *C. eugenioides* distributions may have been more widespread in higher latitudes and could have overlapped with the current *C. arabica* distribution in habitats that were suitable for all three species. In fact, *C. canephora* was probably able to find suitable habitats in Ethiopia in the recent past (mid‐Holocene, ~6000 years before present, R. Tournebize, data not shown). The climatic changes could have reduced the diploid distributions to their current locations. As a consequence, the birthplace of *C. arabica* could possibly be not only in Ethiopia but also in the entire region (South Sudan, Uganda, North Kenya) followed either by migration to present‐day Ethiopia or by survival in that region alone.

Due either to past habitat shift in Eastern Africa or to the colonization of new peripheral geographical areas, the presence of *C. arabica* in different environments and a reduction in diploid competitors would have increased the divergence of initially con‐specific populations and eventually would have given rise to the *C. arabica* speciation. *C. arabica's* self‐fertility would have further contributed to its genetic isolation.

## Conclusion

The availability of our Coffee8.5K to the Coffee community provides an opportunity to generate high‐throughput genotyping data on different types of genetic and breeding populations for accelerating genetic diversity, high‐resolution trait mapping and breeding applications. It will help in identifying the allelic diversity present in the wild relatives of the two cultivated species, offering a clue to the transferability of beneficial alleles to *C. canephora* and/or to *C. arabica*.

Thanks to our stringent selection of genome‐wide distributed and informative SNPs, our array is an efficient tool for fingerprinting. As an application, detecting admixtures up to individual levels has allowed us to assign the Arabica C^a^ subgenome to its closest present‐day *C. canephora* relatives. Our array is definitely a powerful tool for quality control and traceability of all merchant coffees.

## Materials and methods

### Plant material

Accessions used to design the Coffee8.5K array (12 *C. canephora* and 5 *C. arabica*) were chosen to obtain an array for optimized use in mapping and genetic diversity analyses in coffee trees (Table S2).

SNP calling was generated from two Discovery Panels. The *C. arabica* Discovery Panel included five genotypes whose genome sequences were kindly provided by the Arabica Coffee Genome Consortium (ACGC): the parents of the Arabica mapping population (Ar8 and Ar36B), two cultivated varieties (Mundo Novo and Bourbon) and Et39, which is a spontaneous *C. arabica* dihaploid, that is with only one set of chromosomes from each subgenome (see Figure 4b). The *C. canephora* Discovery Panel included 12 genotypes of *C. canephora* belonging to the six genetic groups (Pégard *et al*., 2014).

Coffee8.5K array SNP segregation was monitored in the Robusta BP409 X Q121 and the Arabica Ar8 X Ar36B mapping populations (Mapping Panels, Table S2).

To evaluate the transferability and genotyping performance of the Coffee8.5K array, we genotyped a Diversity Panel of six *C. eugenioides,* the *C. arabica* dihaploid Et39, 16 wild and cultivated *C. arabica* and 27 *C. canephora* accessions representative of the *C. canephora* diversity groups as previously defined with SSR markers (Gomez *et al*., 2009; Pégard *et al*., 2014) (Table S2), as well as uncharacterized individuals from Angola and DRC.

### SNP selection and array design

#### SNP selection and array characterization

We performed SNP calling and SNP selection thanks to the genome resequencing of the five accessions of *C. arabica* kindly provided by the Arabica Coffee Genome Consortium, ACGC (de Kochko, 2018) and of 12 *C. canephora* accessions (this study). SNP calling was performed by mapping the resequencing reads to the reference genome (Denoeud *et al*., 2014) using the NGS workflow manager TOGGLe (http://toggle.southgreen.fr, (Monat *et al*., 2015; Tranchant‐Dubreuil *et al*., 2018)).

All identified SNPs were filtered using custom Perl scripts to select bi‐allelic SNPs of high‐quality (QUAL > 40) and SNP positions with suitable flanking sequences (60 bp on both sides with no variation). Accessions used for SNP discovery and SNP selection criteria were chosen to design an array for optimized use in mapping and genetic diversity analyses in coffee trees.

#### SNPs from *C. arabica*

Screening for *C. arabica* SNPs was conducted on the Arabica Discovery Panel. Set 1, represented by the two wild Ethiopian parents of the Arabica mapping population, Ar8 and Ar36B, together with the dihaploid Et39. We identified 8 208 891 raw SNPs (Set 1, Figure 1), whereas Set 2, represented by Mundo Novo, Bourbon and Et39 sequences generated 10 063 130 raw SNPs (Set 2, Figure 1). Since any SNP scored as heterozygous in Et39 represents polymorphism between the two subgenomes (Figure 4b), to minimize these polymorphisms and to maximize the proportion of true‐allelic SNPs within the subgenome, the following filters were applied to both sets: (i) we removed the polymorphic SNPs in Et39; (ii) we selected SNPs that were homozygous for different alleles in at least two different *C. arabica* accessions; (iii) we considered SNPs spaced with a minimum distance of 40 kb.

#### SNPs from *C. canephora*

The Discovery Panel of 12 *C. canephora* genotypes was used for SNP discovery. To build a reliable and balanced genotyping array, accessions belonging to the six genetic groups (Pégard *et al*., 2014) were selected.

The analysis of the sequencing data yielded a total of 23 590 929 raw SNPs. The following sets were defined when choosing SNPs for microarray design (Figure 1): (i) a set of SNPs were selected on 149 candidate genes with functions related to cup quality (Privat *et al*., 2008), secondary metabolism (Denoeud *et al*., 2014; Lepelley *et al*., 2007, 2012) and drought tolerance as characterized and defined by their gene models on the reference genome (Denoeud *et al*., 2014) (Table S1); (ii) two sets of SNPs of equivalent size selected either on coding or non‐coding regions.

To minimize the impact of SNP ascertainment bias on downstream analyses, only SNPs found in at least two genotypes within the surveyed genotypes were chosen (allelic representation).

### Genotyping assay

#### DNA extraction

Total DNA was extracted from adult leaves using DNeasy Plant Maxi Kit (Qiagen, Hilden, Germany) following the manufacturer's instructions. Final DNA concentration was determined with the Quant‐iT™ PicoGreen^®^ dsDNA Assay Kit (Molecular Probes, Eugene, OR, USA) using the LightCycler^®^ 480 (Roche Diagnostics, Rotkreuz, Switzerland). The DNA quality control was performed according to the Agilent Genomic DNA ScreenTape assay with the Agilent 2200 TapeStation system. Templates were diluted or concentrated to obtain a concentration of 50 ng/μL as required by Illumina for the amplification step.

#### KASPar™ genotyping

The KASPar™ Genotyping System from LGC Genomics^®^ is a competitive allele‐specific dual FRET‐based assay for SNP genotyping (Cuppen, 2007). Among the 9824 SNPs selected for the SNP array design, a subset of 50 single‐nucleotide polymorphism sequences (Figure 1c) distributed across the genome were submitted to LGC Genomics for a KASPar assay design based on the SNP locus‐flanking sequence. The genotyping was conducted at LGC Genomics following their own protocol and the genotype calling was conducted with SNPviewer (LGC Genomics, Hoddesdon, UK).

#### SNP synthesis and calling

The 9827 selected SNPs were submitted to the Illumina Assay Design Tool for design score calculation (www.illumina.com) and 8580 of them were successfully synthesized by Illumina manufacturing processes. The SNP genotyping assay was performed on an Illumina^®^ Infinium HD iSelect Custom Genotyping Array according to the standard Illumina's protocol, using 200 ng of genomic DNA per sample. The extension and staining steps were operated on a Tecan Evo‐150 liquid‐handling robot. Fluorescence intensities were read with Illumina^®^ iScan Control software and allele calling was carried out using the Genome Studio v2.0 software (Illumina Inc., San Diego, CA, USA). All data were visually inspected and manually re‐scored if any errors were evident in the calling of the SNP clusters by the default algorithm. Reproducibility error rates were calculated between the control sample and SNP replicates.

### SNP array applications

To evaluate the performance of the Coffee8.5K array, we genotyped the *C. canephora* and *C. arabica* mapping populations, a set of six *C. eugenioides,* 17 *C. arabica* individuals including the dihaploid Et39 and a Diversity Panel of 27 *C. canephora* individuals representative of the species diversity groups, previously genotyped with microsatellite markers with known genetic group assignation (Gomez *et al*., 2009; Pégard *et al*., 2014), as well as uncharacterized individuals from Angola and DRC.

#### Mapping population and linkage analysis

Two mapping populations were used, one Robusta progeny based on a F_1_ cross between BP409 (Congolese hybrid) and Q121 (Conilon‐type derived accession) comprising 93 individuals and an Arabica F_2_ segregation population (138 individuals) originating from a cross between two wild Ethiopian Arabica: Ar8 and Ar36B (see Mapping Panels, Table S2).

The Robusta (BP409xQ121) genetic linkage map was built using JoinMap^®^4.1 software (Van Ooijen, 2011) with the regression mapping algorithm and the grouping and recombination settings as described in Mérot‐L'Anthoëne *et al*. (2014). The Kosambi mapping function was used to convert recombination frequencies into map distance and the linkage maps were drawn using MapChart 2.3 (Plant Research International, Wageningen, Netherlands).

To facilitate the access and use of genome‐wide SNPs for geneticists and breeders, we implemented the MoccaDB v 2.0 database initially published in Plechakova *et al*. (2009) with new modules to include information and search tools related to SNPs. The query results can be visualized as text format in tables displayed on interfaces, the maps can be viewed and compared as graphics using the C‐Map (http://gmod.org/wiki/CMap) and in a genome browser (Figure S2).

#### Linkage disequilibrium

Linkage disequilibrium analysis was performed using the whole‐genome resequencing set of *C. canephora* Discovery Panel (*n *=* *12). The linkage disequilibrium decay curve was calculated as *r* ^2^, the squared value of the correlation coefficient of the allelic states of two given polymorphic loci, against biallelic SNPs with MAF > 0.10 and with <10% missing genotypes (excluding the virtual pseudo‐chromosome 0 of unanchored sequences) using the PopLDdecay software (Xu *et al*., 2012).

#### Transferability to related species (*C. arabica*,* C. eugenioides* and *C. canephora*) and diversity analyses

Diversity analyses were performed to confirm the resolution capacity of this array system between accessions on each of the Diversity Panels (Table S2). The number of usable SNP markers for each of the three species was scored in relation to the number of total scorable loci, whereas the level of polymorphic markers was calculated for each species in relation to their number of usable markers. Since Et39 is a dihaploid with only one set of chromosomes from each subgenome, any SNP scored as heterozygous in Et39 represents polymorphism between the two subgenomes (Figure 4b). To optimize the estimation of within subgenomes polymorphism, the proportion of polymorphic SNPs within the set of 16 *C. arabica* was thus calculated after removing the heterozygous SNPs or SNPs with missing genotypes in Et39 (Table 1).

Population structure within the *C. canephora* subset was analysed using an unsupervised clustering algorithm, sNMF (Frichot *et al*., 2014) using the 27 *C. canephora* accessions representative of the species diversity including the parents of the Robusta mapping population. The most likely number of genetic clusters (*K*) was determined as the one minimizing the cross‐entropy criterion, for *K* ranging between 1 and 10 (with 20 estimation runs per *K*).

Two neighbour‐joining dendrograms were constructed based on the Euclidean distances between genotypes, one for the three species together (4095 polymorphic markers without missing data) and the other one for the *C. arabica* individuals (595 intra‐specific polymorphic markers without missing data). The stability of each node was evaluated using 100 bootstraps by blocks of 100 SNPs (R package *ape*) (Popescu *et al*., 2012).

#### Assignation of the arabica subgenomes

*Coffea arabica* contains two diploid genomes C^a^ and E^a^ that originated from two different wild diploid ancestor species, *C. canephora* and *C. eugenioides* (Lashermes *et al*., 1999). The similarity of the *canephora*‐derived subgenome C^a^ to current *C. canephora* genotypes and *eugenioides*‐derived subgenome E^a^ to current *C. eugenioides* accessions was evaluated based on the representative Diversity Panels of both species. Based on 4258 shared SNPs without missing data, Identity‐by‐state (IBS) distances were computed between each of the 17 *C. arabica* accessions with each individual without admixture of the two related species. We used haploid genotype downscaling to mitigate the impact of the polymorphic SNPs in *C. arabica* that cannot be precisely assigned to one or the other related species. Haploid genotypes were randomly drawn for each SNP and each accession over 200 runs. The IBS distances were averaged over all runs for each *C. arabica* accession. The average IBS distances and their standard deviations were then calculated over all 17 *C. arabica* accessions.

## Conflicts of interest

The authors declare no conflicts of interest.

## Supporting information


**Figure S1** Linkage disequilibrium

**Figure S2** MoccaDB v2 database screenshots

**Figure S3** *C. arabica* neighbour‐joining tree

**Table S1** List of the Candidate Genes with their corresponding SNP markers (Locus ID and location of each original SNP is provided according to Denoeud *et al*. (2014) (2014) annotation)

**Table S2** List of accessions‐ List of accessions and their use: Discovery/Diversity/Mapping panel‐ Geographical location of the *C. arabica* samples used in this study overlaid on the Guillaumet (1978) map‐ Location of analysed *C. eugenioides* individuals on the microsatellite neighbour‐joining tree

**Table S3** SNP information (origin, genome location, sequence, polymorphism)

**Table S4** Summary table of the historical definition of *C. canephora* genetic groups with the references and the marker types in use.
