# Supplementary figures and images for: Development and evaluation of a genome‐wide Coffee 8.5K SNP array and its application for high‐density genetic mapping and for investigating the origin of Coffea arabica L
Source: Plant Biotechnol J. 2019 Feb 4;17(7):1418–30. doi: 10.1111/pbi.13066 (PMC6576098; doi:10.1111/pbi.13066)

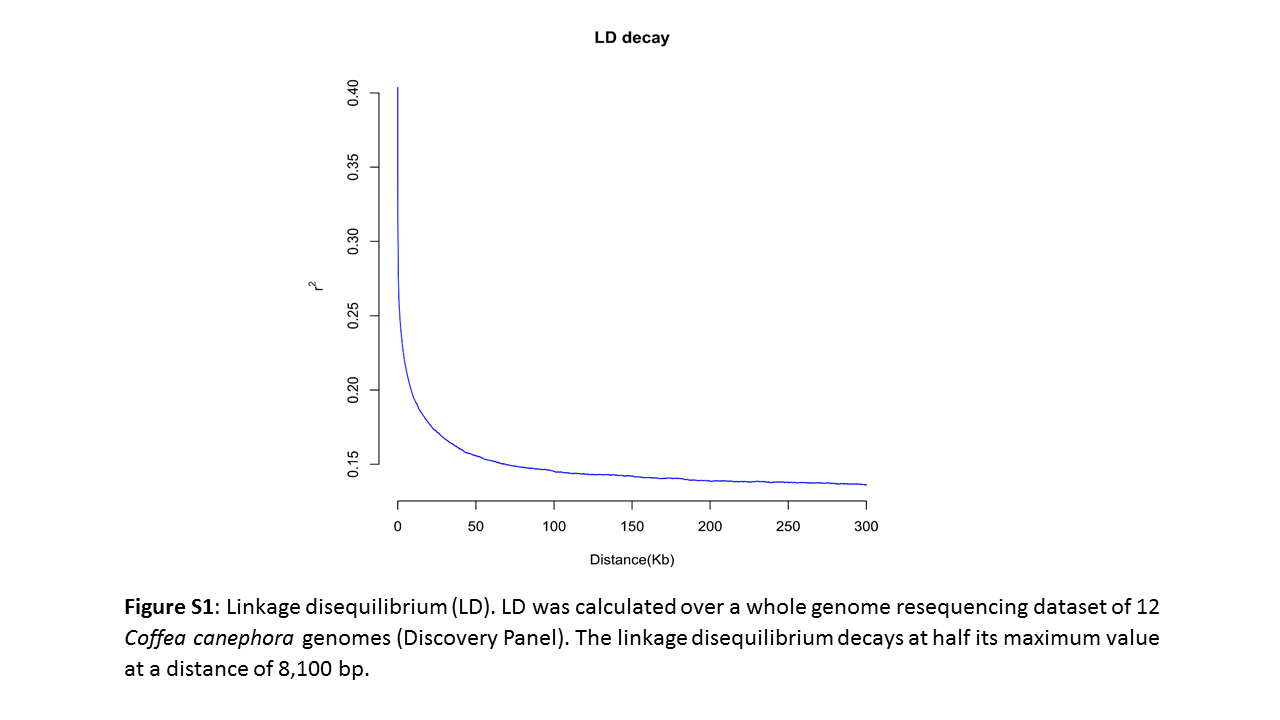

Supplement: Supplementary file 1 — Figure S1 Linkage disequilibrium [file PBI-17-1418-s006.png]

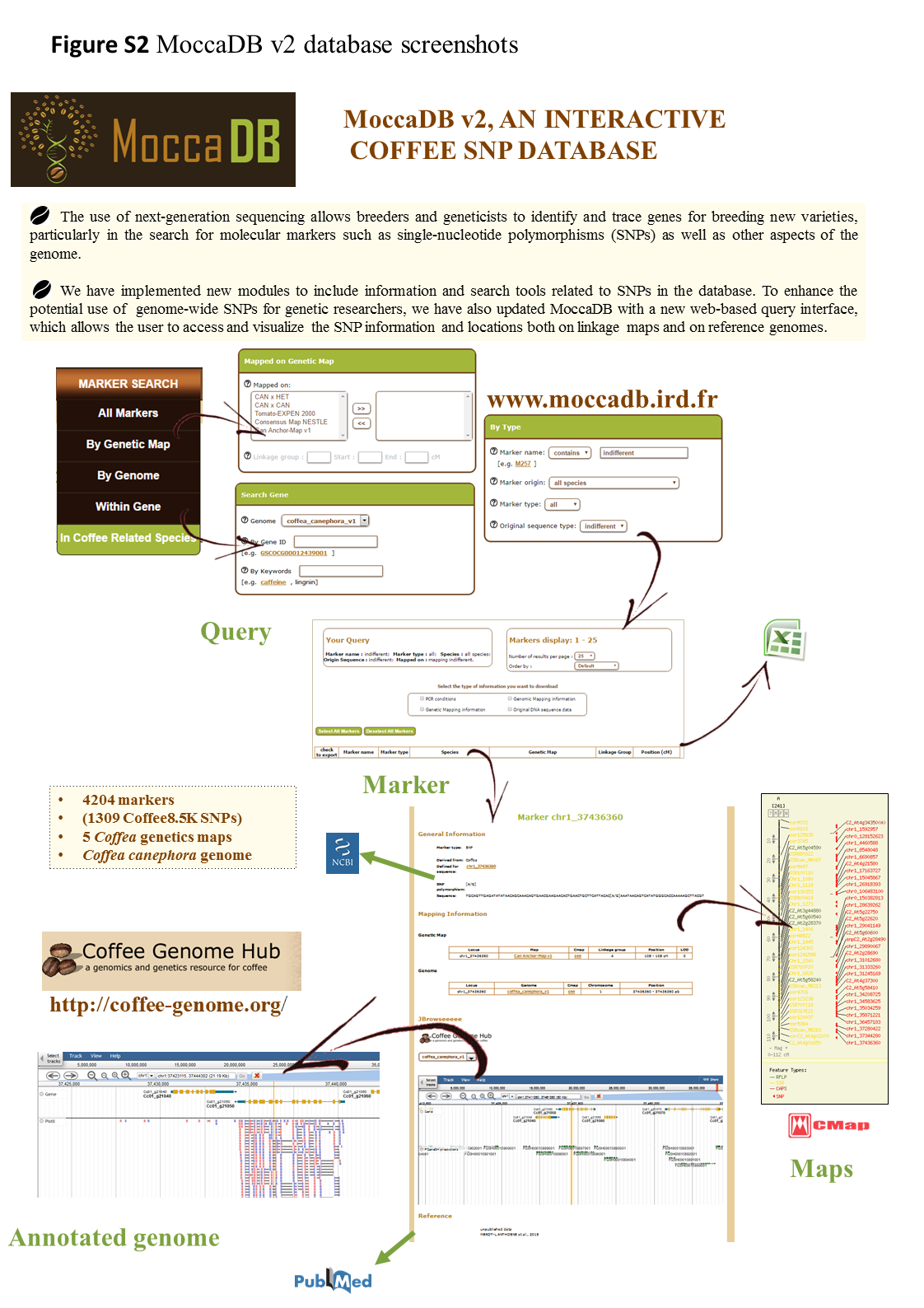

Supplement: Supplementary file 2 — Figure S2 MoccaDB v2 database screenshots [file PBI-17-1418-s005.png]

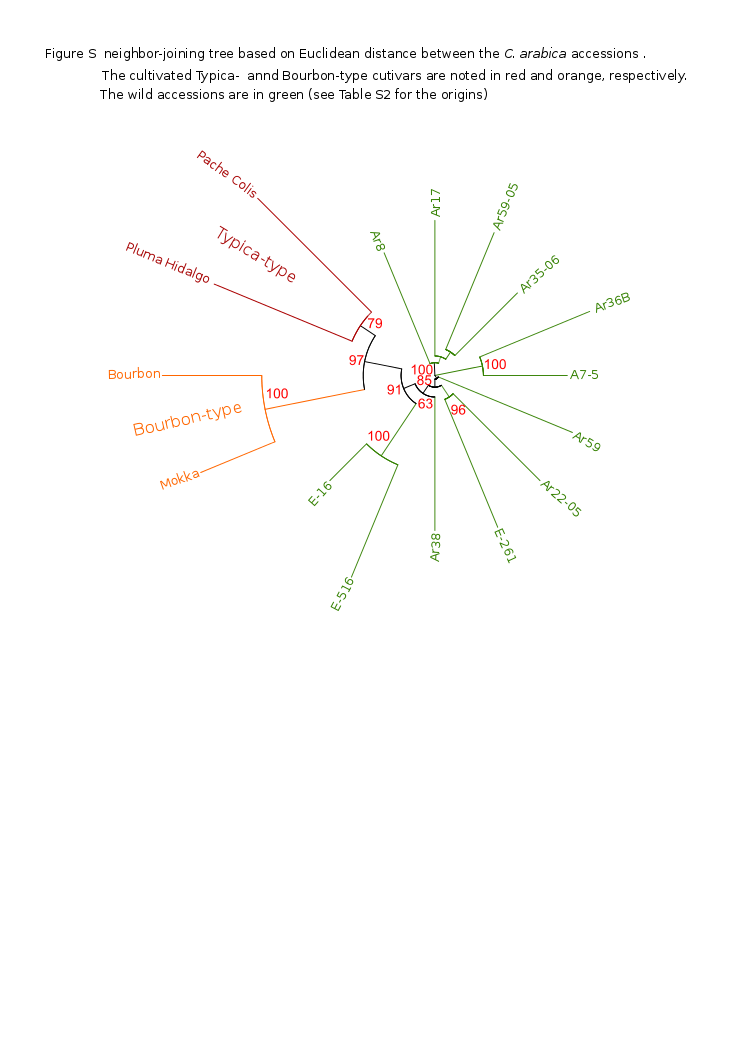

Supplement: Supplementary file 3 — Figure S3 C. arabica neighbour‐joining tree [file PBI-17-1418-s004.png]
