# Supplementary material for: Development and evaluation of a genome‐wide Coffee 8.5K SNP array and its application for high‐density genetic mapping and for investigating the origin of Coffea arabica L
Source: Plant Biotechnol J. 2019 Feb 4;17(7):1418–30. doi: 10.1111/pbi.13066 (PMC6576098; doi:10.1111/pbi.13066)
Supplement: Supplementary file 7 — Table S4 Summary table of the historical definition of C. canephora genetic groups with the references and the marker types in use. [file PBI-17-1418-s001.docx]

Table S4 Summary table of the historical definition of *C. canephora* genetic groups with the references and the marker types in use

| **Study** | **Berthaud, 1986** | **Montagnon et al., 1992** | **Cubry et al., 2008**  **Musoli et al. 2009** | **Dussert et al, 1999**  **Gomez et al, 2009** | **PRESENT**  **STUDY** | **Geographic origin** | |
| --- | --- | --- | --- | --- | --- | --- | --- |
|  | **Isozymes** | **Isozymes** | **SSR** | **RFLP, SSR** | **SNP** | Wild | Cultivated |
| **Genetic groups** | Guinean | Guinean | Guinean | D | **D** | Guinea, Côte d’Ivoire | Guinea, Côte d’Ivoire |
|  | Congolese | SG1 | SG1 | A | **A** | North of Congo, South of Cameroon | « Niaouli », « Conilon »  Togo, Côte d'Ivoire |
|  |  | SG2 | SG2 | B | **B** | East of Central Afr. Rep. |  |
|  |  |  |  | E | **E** | Democr. Rep of the Congo, Cameroon | Guinea |
|  |  | Not included | C | C | **C** | West of Central Afr. Rep., Cameroon |  |
|  | Not included |  | Ug | Not included | **O** | Uganda, South Sudan |  |
|  |  |  | Not included | Not included | **R** | **South of the Democr. Rep of the Congo** |  |
|  |  |  |  |  | **A** | **North and West of Angola** |  |
